
JOURNAL INFORMATION
==============================
NLM Title Abbreviation: Orthop Surg
Journal ID: Orthop Surg
NLM Journal ID: 101501666
Journal ID: OS

Orthopaedic Surgery

ISSN: 1757-7853
EISSN: 1757-7861
Publisher: Wiley

ARTICLE INFORMATION
==============================
PMCID: PMC11474567
DOI: 10.1111/os.13345
Article ID: OS13345
Article version: 1
Subjects: Issue Information, Issue Information

Issue Information

Electronic publication date: 2023 Nov 2
Publication date: 2023 Nov
Volume: 15
Issue: 11
First page: 2741
Last page: 2742
Copyright: © 2023 The Authors. Orthopaedic Surgery published by Tianjin Hospital and John Wiley & Sons Australia, Ltd.
License: This is an open access article under the terms of the http://creativecommons.org/licenses/by/4.0/ License, which permits use, distribution and reproduction in any medium, provided the original work is properly cited.
License URL: https://creativecommons.org/licenses/by/4.0/

Figure count: 0
Table count: 0
Page count: 2

source-schema-version-number: 2.0
cover-date: November 2023
details-of-publishers-convertor: Converter:WILEY_ML3GV2_TO_JATSPMC version:6.4.9 mode:remove_FC converted:15.10.2024

==============================

Volume 15 • Issue 11 • November 2023 • ISSN 1757-7861

### Orthopaedic Surgery

Open Access

REVIEW ARTICLES

2743 OPEN SURGICAL TREATMENTS OF OSTEOPOROTIC VERTEBRAL COMPRESSION FRACTURES

2749 STRATEGIES FOR CARTILAGE REPAIR IN OSTEOARTHRITIS BASED ON DIVERSE MESENCHYMAL STEM CELLS-DERIVED EXTRACELLULAR VESICLES

2766 RESULTS OF MEDIAL PATELLOFEMORAL LIGAMENT RECONSTRUCTION WITH AND WITHOUT TIBIAL TUBERCLE TRANSFER IN PATELLAR INSTABILITY: A SYSTEMATIC REVIEW AND META-ANALYSIS

CLINICAL ARTICLES

2777 IS A PREOPERATIVE MRI SCAN NECESSARY FOR ACUTE ACHILLES TENDON RUPTURE?

2786 MICRO ELECTROMECHANICAL SYSTEM NAVIGATION ASSISTS FEMORAL EXTRAMEDULLARY ALIGNMENT OSTEOTOMY IN TOTAL KNEE ARTHROPLASTY: A SINGLE-BLIND RANDOMIZING STUDY

2794 FINITE ELEMENT PARAMETRIC DESIGN OF HALLUX VALGUS ORTHOSIS BASED ON ORTHOGONAL ANALYSIS

2805 IS SLIDING COMPRESSION NECESSARY FOR INTRAMEDULLARY NAILING FIXATION OF AO/OTA TYPE A3.3 INTERTROCHANTERIC FRACTURE?

2814 THE GENETIC CAUSAL ASSOCIATION BETWEEN EDUCATIONAL ATTAINMENT AND RISK OF 12 COMMON MUSCULOSKELETAL DISORDERS: A TWO-SAMPLE MENDELIAN RANDOMIZATION

2822 RISK FACTORS FOR SIGNIFICANT INTRAOPERATIVE BLOOD LOSS DURING ANTERIOR CERVICAL DECOMPRESSION AND FUSION FOR DEGENERATIVE CERVICAL DISEASES

2830 A COMPARISON OF CORPECTOMY ACDF HYBRID PROCEDURES WITH NANO-HYDROXYAPATITE/POLYAMIDE 66 CAGE AND TITANIUM MESH CAGE FOR MULTI-LEVEL DEGENERATIVE CERVICAL MYELOPATHY: A STEPWISE PROPENSITY SCORE MATCHING ANALYSIS

2839 LUMBAR DISC HERNIATION WITH CONTRALATERAL SYMPTOMS: A CASE-SERIES OF 11 PATIENTS AND LITERATURE REVIEW

2848 THE INFLUENCE OF COMPENSATORY PELVIC TILT ON PATIENT-REPORTED OUTCOME MEASUREMENTS IN FAI PATIENTS WHO RECEIVED ARTHROSCOPIC TREATMENT

2855 COMPARISON OF COMPUTER-ASSISTED NAVIGATION AND 3D PRINTED PATIENT-SPECIFIC TEMPLATE FOR THE ILIOSACRAL SCREW PLACEMENT

2864 CERAMIC-ON-CERAMIC OR METAL-ON-POLYETHYLENE: THE BEARING OF CHOICE AFTER CERAMIC COMPONENT FRACTURE IN TOTAL HIP ARTHROPLASTY ALONG WITH CONCISE FOLLOW-UP OF THE PREVIOUS COHORT

2872 SPINE SURGERY WITH ELECTRONIC CONDUCTIVITY DEVICE: A PROSPECTIVELY MULTICENTER RANDOMIZED CLINICAL TRIAL AND LITERATURE REVIEW

2881 NOT ALL OSTEOPHYTES ARE LOCATED ON THE RIGHT SIDE OF THE VERTEBRAE IN DIFFUSE IDIOPATHIC SKELETAL HYPEROSTOSIS: A QUANTITATIVE ANALYSIS IN RELATION TO THE POSITION OF AORTA

2889 COMPARISON BETWEEN THREE-DIMENSIONAL PRINTED TITANIUM AND PEEK CAGES FOR CERVICAL AND LUMBAR INTERBODY FUSION: A PROSPECTIVE CONTROLLED TRIAL

2901 CERVICAL DISC ARTHROPLASTY FOR THE TREATMENT OF NONCONTIGUOUS CERVICAL DEGENERATIVE DISC DISEASE: RESULTS OF MID- TO LONG-TERM FOLLOW-UP

RESEARCH ARTICLES

2911 VALIDATION OF A WEARABLE SYSTEM FOR LOWER EXTREMITY ASSESSMENT

2918 THE EFFECT OF HALO-PELVIC TRACTION ON BONE MINERAL DENSITY OF VERTEBRAE AND CORRESPONDING RISK FACTORS

2927 A NOVEL SEMI-CANNULATED SCREW ENHANCED BONE CEMENT AUGMENTATION AND PULLOUT STRENGTH IN POSTERIOR CERVICAL LATERAL MASS SCREW FIXATIONS: AN *IN VITRO* BIOMECHANICAL AND MORPHOLOGICAL STUDY

2937 STABILITY OF THREE-DIMENSIONAL PRINTED CUSTOM-MADE METAPHYSEAL CONE FOR TIBIAL BONE DEFECTS RECONSTRUCTION: A FINITE ELEMENT ANALYSIS AND BIOMECHANICAL STUDY

2947 ANALYSIS OF ZINC AND STROMAL IMMUNITY IN DISUSE OSTEOPOROSIS: MENDELIAN RANDOMIZATION AND TRANSCRIPTOMIC ANALYSIS

2960 METHODS OF IMMOBILIZATION AFTER ACHILLES TENDON RUPTURE REPAIR: A COMPARATIVE STUDY IN RAT MODEL

OPERATIVE TECHNIQUES

2966 DUAL PARALLEL INTRAMEDULLARY K-WIRES FOR ARTHRODESIS OF THE FINGER DISTAL OR THUMB INTERPHALANGEAL JOINTS

2974 CLINICAL ANALYSIS OF THE FROSCH APPROACH IN THE TREATMENT OF POSTEROLATERAL TIBIAL PLATEAU FRACTURES COMBINED WITH LATERAL TIBIAL PLATEAU FRACTURES

2985 AUTOLOGOUS COSTAL OSTEOCHONDRAL TRANSPLANTATION FOR CYSTIC OSTEOCHONDRAL LESIONS OF THE TALUS: FEASIBLE AND EFFECTIVE

CASE REPORTS

2993 LEVODOPA-CARBIDOPA INTESTINAL GEL INJECTION FOR PATIENT WITH SEVERE PARKINSON'S DISEASE FOLLOWED BY TOTAL HIP ARTHROPLASTY: A CASE REPORT AND LITERATURE REVIEW

3000 COMPUTER-AIDED DESIGN AND 3D-PRINTED PERSONALIZED STEM-PLATE COMPOSITE FOR PRECISION REVISION OF THE PROXIMAL HUMERUS ENDOPROSTHETIC REPLACEMENT: A TECHNIQUE NOTE

3006 A CASE OF TKA FAILURE IN PATIENT WITH PRIMARY HYPERPARATHYROIDISM

WILEY

CHINESE ORTHOPAEDIC ASSOCIATION
中华医学会骨科学分会

View this journal online at wileyonlinelibrary.com/journal/os

### Orthopaedic Surgery

**Editor-in-Chief**
Gui-xing Qiu, *Beijing, China*
Francis John Hornicek, *Boston, USA*

**Associate Editor**
Yong-cheng Hu, *Tianjin, China*
Thomas Blattert, *Leipzig, Germany*
Douglas Letson, *Tampa, USA*
Xin-long Ma, *Tianjin, China*
Ling Qin, *Hong Kong, China*

**Editorial Board**
Giovanni Alessi, *Gent, Belgium*
Haluk Berk, *Izmir, Turkey*
Thomas Blattert, *Leipzig, Germany*
Mathis Bostrom, *New York, USA*
Kao-wha Chang, *Taiwan, China*
Edmund YS Chao, *Baltimore, USA*
Liao-bin Chen, *Wuhan, China*
Yan-xi Chen, *Shanghai, China*
Kenneth MC Cheung, *Hong Kong, China*
Thomas L. Clemens, *Birmingham, USA*
Benny Thellefsen Dahl, *Copenhagen, Denmark*
Lian-fu Deng, *Shanghai, China*
Jean Destandau, *Bordeaux, France*
Jian Dong, *Shanghai, China*
Shun-wu Fan, *Hangzhou, China*
David Fang, *Hong Kong, China*
Shi-jun Gao, *Shijiazhuang, China*
Wei Guo, *Beijing, China*
Yong Hai, *Beijing, China*
Alan R. Hargens, *San Diego, USA*
Francis John Hornicek, *Boston, USA*
William C. Horton, *Atlanta, USA*
John Hsiang, *Seattle, USA*
Yong-cheng Hu, *Tianjin, China*
Qing Jiang, *Nanjing, China*
Paul Jutte, *Groningen, Netherlands*
Jean Pierre Kalala, *Gent, Belgium*
Choon-Ki Lee, *Seoul, Korea*
Jean Jacque Lehuec, *Bordeaux, France*
Lawrence G. Lenke, *Seattle, USA*
Douglas Letson, *Tampa, USA*
Kwok Sui Leung, *Hong Kong, China*
Jian-hao Lin, *Beijing, China*
Bao-ge Liu, *Beijing, China*
Wei-hee Lo, *Taiwan, China*
William Weijia Lu, *Hong Kong, China*
Keith DK Luk, *Hong Kong, China*
Zong-ping Luo, *Suzhou, China*
Cyril Mauffrey, *Denver, USA*
Wei-hu Ma, *Ningbo, China*
Xin-long Ma, *Tianjin, China*
Ling Qin, *Hong Kong, China*
Gui-xing Qiu, *Beijing, China*
Axel Reinhardt, *Potsdam, Germany*
Pu-yi Sheng, *Guangzhou, China*
Harry L Shufflebarger, *Miami, USA*
David Stulberg, *Chicago, USA*
Paul Sung, *San Diego, USA*
Paul Unwin, *Leicester, UK*
Luc F. De Waele, *Gent, Belgium*
Fei Wang, *Shijiazhuang, China*
Wei-jun Wang, *Nanjing, China*
James N. Weinstein, *Chicago, USA*
Hee-Kit Wong, *Singapore*
Su-jia Wu, *Nanjing, China*
Jian-ru Xiao, *Shanghai, China*
Hui-lin Yang, *Suzhou, China*
Tzai-chiu Yu, *Taiwan, China*
Xi-jie Yu, *Chengdu, China*
Hansen A. Yuan, *New York, USA*
Hong-wen Zhu, *New York, USA*

**Address for Editorial Correspondence:** Editorial Board of *Orthopaedic Surgery*, Tianjin Hospital, 122 Munan Road, Tianjin 300050, China (email: orthopaedicsurgery@126.com; telephone: +86 22 2833 4734; fax: +86 22 2824 1184).

**Disclaimer:** The Publisher, Tianjin Hospital and Editors cannot be held responsible for errors or any consequences arising from the use of information contained in this journal; the views and opinions expressed do not necessarily reflect those of the Publisher, Tianjin Hospital and Editors, neither does the publication of advertisements constitute any endorsement by the Publisher, Tianjin Hospital and Editors of the products advertised.

**Copyright © 2023 The Authors. Orthopaedic Surgery published by Tianjin Hospital and John Wiley & Sons Australia, Ltd**

**Copyright and Copying (in any format)**
Copyright © 2023 The Authors. Orthopaedic Surgery published by Tianjin Hospital and John Wiley & Sons Australia, Ltd. All rights reserved. No part of this publication may be reproduced, stored or transmitted in any form or by any means without the prior permission in writing from the copyright holder. Authorization to copy items for internal and personal use is granted by the copyright holder for libraries and other users registered with their local Reproduction Rights Organisation (RRO), e.g. Copyright Clearance Center (CCC), 222 Rosewood Drive, Danvers, MA 01923, USA (www.copyright.com), provided the appropriate fee is paid directly to the RRO. This consent does not extend to other kinds of copying such as copying for general distribution, for advertising or promotional purposes, for republication, for creating new collective works or for resale. Permissions for such reuse can be obtained using the RightsLink “Request Permissions” link on Wiley Online Library. Special requests should be addressed to: permissions@wiley.com

For submission instructions, subscription and all other information visit: http://wileyonlinelibrary.com/journal/os

ISSN 1757-7861 (Online)

OS.JEB.November23
